# Clinical outcomes following medial meniscus posterior root repairs: A minimum of 5‐year follow‐up study

**DOI:** 10.1002/jeo2.70262

**Published:** 2025-05-07

**Authors:** Yuki Okazaki, Kazuhisa Sugiu, Yusuke Kamatsuki, Masanori Tamura, Koki Kawada, Tsubasa Hasegawa, Takayuki Furumatsu

**Affiliations:** ^1^ Department of Orthopaedic Surgery Okayama University Graduate School of Medicine, Dentistry, and Pharmaceutical Sciences Okayama Japan; ^2^ Department of Orthopaedic Surgery Okayama Red Cross Hospital Okayama Japan; ^3^ Department of Orthopaedic Surgery Okayama Saiseikai General Hospital Okayama Japan

**Keywords:** clinical outcome, medial meniscus posterior root tear, mid‐term follow‐up, survival rate, transtibial pullout repair

## Abstract

**Purpose:**

This study assessed the clinical outcomes of the FasT‐Fix dependent modified Mason‐Allen suture (F‐MMA) and two simple stitches (TSS) on mid‐term postoperative outcomes following medial meniscus (MM) posterior root repair.

**Methods:**

Forty‐three patients who underwent transtibial pullout repair for MM posterior root tear (PRT) between November 2016 and September 2018 were initially enrolled. Patients with a femorotibial angle ≤ 180°, Kellgren–Lawrence grade of 0–2, and modified Outerbridge grade I or II cartilage lesions were included. The Lysholm, Tegner activity, International Knee Documentation Committee score, pain visual analogue scale and Knee injury and Osteoarthritis Outcome scores were assessed as clinical outcomes. Conversion surgery to knee arthroplasty was considered as the endpoint. Surgeries other than second‐look arthroscopy and plate or screw removal were also recorded.

**Results:**

The mean follow‐up period was 5.9 years. All evaluated 5‐year postoperative clinical outcomes were significantly improved compared to the preoperative outcomes (*p* < 0.001). Both the F‐MMA and TSS significantly improved all clinical scores at 5 years postoperatively in patients with MMPRT, whereas the F‐MMA and TSS groups showed no significant differences in the pre‐ and postoperative clinical scores. None of the patients required ipsilateral knee arthroplasty during the follow‐up, and the survival rate after pullout repair was 100%. However, the progression of osteoarthritis could not be completely suppressed, although there were no Kellgren–Lawrence grade 4 cases. The rate of subsequent knee‐related surgical treatment was 11.6% in pullout‐repaired knees, including arthroscopic debridement for arthrofibrosis with a limited range of motion, an additional all‐inside suture repair and partial meniscectomy.

**Conclusion:**

Both F‐MMA and TSS pullout repairs yielded satisfactory clinical outcomes in patients with MMPRT with a mean follow‐up of 5.9 years, and no conversion to knee arthroplasty was required. Further follow‐up is warranted to assess long‐term survival rates.

**Level of Evidence:**

Level III.

**Abbreviations**
ADL: activities of daily living
BMI: body mass index
F‐MMA: FasT‐Fix‐dependent modified Mason‐Allen
HTO: high tibial osteotomy
IKDC: International Knee Documentation Committee
KL: Kellgren–Lawrence
KOOS: Knee injury and Osteoarthritis Outcome Scores
MCID: minimal clinically important difference
MM: medial meniscus
OA: osteoarthritis
PASS: patient‐acceptable symptomatic state
PR: posterior root
PRT: posterior root tear
QoL: quality of life
Sport/Rec: sports and recreational function
TSS: two simple stitches
VAS: visual analogue scale

## INTRODUCTION

Medial meniscus (MM) posterior root tear (PRT) often occurs in middle‐aged women, especially with painful popping episodes while descending stairs [11, 14], leading to abnormal tibiofemoral joint biomechanics, MM posteromedial extrusion during knee flexion, and overloading of the articular cartilage due to the inability to convert axial loads into hoop stresses [2, 33, 34, 35, 36]. Although high body mass index (BMI) and varus knee alignment are independent risk factors for this condition, MM posterior root (PR) repair is currently the recommended treatment and is preferred over conservative therapies or partial meniscectomies for MMPRTs based on biomechanical and long‐term follow‐up clinical studies [4, 5, 6, 22, 29, 38].

Varus knee alignment is a factor of poor short‐, medium‐ and long‐term postoperative clinical outcomes in patients with MMPRTs. A varus alignment of >5° is the principal risk factor for this condition [5, 13, 28, 38]. Therefore, MMPRT with moderate‐to‐severe varus knee deformity is typically treated with a high tibial osteotomy (HTO), either independently or in combination with MMPR repair [24, 25, 26, 30]. Recently, MMPR repair combined with HTO has been used to treat MMPRTs with moderate varus knee alignment [12, 20]. A follow‐up >5 years after partial meniscectomy for MMPRT revealed that the radiological progression of osteoarthritis (OA) was more advanced in patients with a knee varus >3° than in those with a neutral alignment ≤ 3° [19]. Although there are long‐term reports of up to 10 years following pullout repair, these reports also include Kellgren–Lawrence (KL) grade 3 OA [6]. Regarding the configuration, the (modified) Mason‐Allen suture has been reported to be superior to two simple stitches (TSS) [7]. However, clinical outcomes following MMPR repair is comparable across different suture configurations [8], and few studies have reported the mid‐ to long‐term outcomes in patients with mild varus alignment without severe knee OA [4].

This study aimed to investigate the mid‐term clinical outcomes following acute MMPR repair using the FasT‐Fix‐dependent modified Mason‐Allen (F‐MMA) suture and TSS in patients with no or mild varus knee alignment, as well as the conversion rate to arthroplasty. We hypothesised that the mid‐term postoperative clinical outcomes following both F‐MMA and TSS suture techniques in these selected patients would be excellent, with an extremely low incidence of conversion to arthroplasty.

## MATERIALS AND METHODS

### Patients

**Figure 1:**
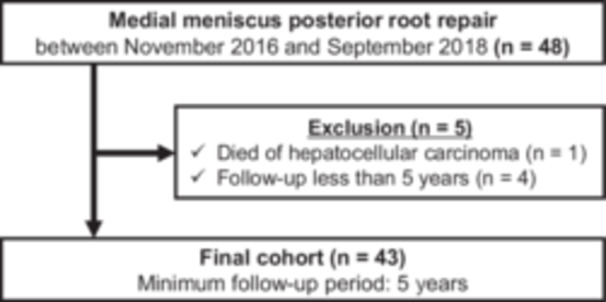
Flowchart of patient recruitment.

This study was approved by the Institutional Review Board (#1857). Written informed consent was obtained from all participants before their participation. The study was conducted in accordance with the principles of the Declaration of Helsinki. A total of 48 consecutive participants who underwent transtibial pullout repair for MMPRTs between November 2016 and September 2018 were initially enrolled (Figure 1).

Inclusion criteria were as follows: (1) arthroscopic transtibial pullout repair of MMPRTs; (2) participants with a femorotibial angle (FTA) ≤ 180°; (3) a KL grade of 0–2; [18] and (4) mild modified Outerbridge grade I or II cartilage lesions [37]. Patients with missing preoperative or minimum 5‐year postoperative clinical outcome data were excluded. Ultimately, 43 patients were included in this study.

Age at the time of surgery, sex, height, weight, BMI, duration from injury to surgery, FTA, and the KL grade were recorded for each patient. The injury date was defined as the date when patients reported experiencing the ‘painful posteromedial popping episode’ [3]. MMPRT types were classified based on tear morphology, as previously described for types 1–5 [23]. The meniscal healing status was evaluated by a senior orthopaedic surgeon during second‐look arthroscopy 1 year after arthroscopic transtibial pullout repair [15]. The semi‐quantitative scoring system was as follows: (1) anteroposterior width of the bridging tissues between the MM posterior horn and root attachment, broad/narrow/filamentous, scored as 4/2/0; (2) stability of the repaired MMPR, good/fair/loose/useless/detached, scored as 4/3/2/1/0; and (3) synovial coverage of the sutures, good/fair/poor, scored as 2/1/0. Meniscal healing was scored on a scale of 0–10 points [9]. An experienced Orthopaedic surgeon performed all the surgeries in this cohort.

### Surgical technique and rehabilitation protocol

**Figure 2:**
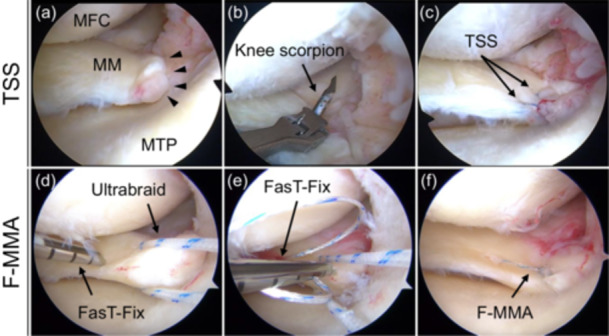
Arthroscopic findings of pullout repair for a medial meniscus posterior root tear (MMPRT) in the left knees. The repair included two simple stitches (TSS, a–c) and a FasT‐Fix dependent modified Mason‐Allen suture (F‐MMA, d–f)**.** (a) MMPRT was confirmed. (b) A No. 2 Ultrabraid suture was passed through the meniscal tissue using a Knee Scorpion suture passer. (c) The TSS configuration was confirmed. (d) The first anchor of the FasT‐Fix was inserted into the meniscal tissue just lateral to the Ultrabraid. (e) The second anchor of the FasT‐Fix was inserted into the meniscal tissue, crossing the Ultrabraid. (f) The F‐MMA configuration was confirmed.

Patients with MMPRTs underwent arthroscopic transtibial pullout repair and postoperative rehabilitation as previously described [10, 21, 32]. Briefly, the F‐MMA or TSS was used to grasp the posterior horn and root. We used a Knee Scorpion suture passer to pass No. 2 Ultrabraid sutures vertically through the meniscal tissue. For F‐MMA, the FasT‐Fix 360 (Smith & Nephew, London, UK) was inserted crossing over the Ultrabraid, while for TSS, only two Ultrabraid sutures were used (Figure 2). Tibial fixation of the sutures was performed using a double‐spike plate (Meira, Aichi, Japan) or a bioabsorbable screw (Smith & Nephew) at 20–45° knee flexion with an initial tension of 20–30 N. Subsequently, the patients were initially kept non‐weight‐bearing with a knee immobiliser for 2 weeks. Between 2 and 4 weeks, knee flexion exercises gradually increased under partial weight‐bearing conditions. After 5 or 6 weeks, the patients were allowed full weight‐bearing and 120° of knee flexion.

### Clinical outcomes

Clinical evaluations were performed at the time of arthroscopic transtibial pullout repair for MMPRT and 5 years postoperatively. The following clinical outcomes were assessed: Lysholm score (0 = worst, 100 = best), Tegner activity score (0 = worst, 10 = best), International Knee Documentation Committee (IKDC) score (0 = worst, 100 = best), pain visual analogue scale (VAS; 0 = no pain, 100 = worst possible pain), and Knee injury and Osteoarthritis Outcome Scores (KOOS), which comprises five subscales: pain, symptoms, activities of daily living (ADL), sports and recreational function (Sport/Rec) and knee‐related quality of life (QOL). In each category, 0 was the worst score and 100 was the best. Conversion to knee arthroplasty was indicated for patients with recurrent or persistent severe knee pain, which was defined as the endpoint. Surgeries other than second‐look arthroscopy and plate or screw removal were also recorded.

### Statistical analysis

Data are presented as mean ± standard deviation. Statistical analyses and power calculations were performed using EZR software (Saitama Medical Center, Jichi Medical University, Tochigi, Japan) [24]. Intragroup differences were compared using the Wilcoxon signed‐rank test. Statistical significance was set at *p* < 0.05. Radiographic findings (KL grade) were assessed by two Orthopaedic surgeons. Interobserver reliability was assessed using ICC and was excellent (0.96).

## RESULTS

**Figure 3:**
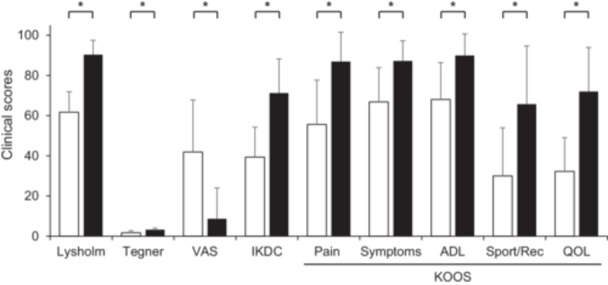
Comparison of preoperative and postoperative clinical scores. All scores significantly improved at final follow‐up period (Wilcoxon signed‐rank test). ADL, activities of daily living; IKDC, International Knee Documentation Committee subjective knee evaluation form; KOOS, Knee Injury and Osteoarthritis Outcome Score; QOL, quality of life; Sport/Rec, sport and recreation function; VAS, pain visual analogue scale *p < 0.001.

At 5 years of follow‐up, there were 15 cases; at 6 years, 20 cases; at 7 years, 6 cases; and at 8 years, 2 cases, with a mean follow‐up of 5.9 years. Table 1 shows patient characteristics at the time of surgery. The mean age, BMI, and FTA were 63.1 ± 7.4 year, 26.5 ± 4.1 kg/m^2^ and 177.5 ± 1.5°, respectively. All evaluated 5‐year postoperative clinical outcomes were significantly improved compared with the preoperative outcomes (*p* < 0.001; Table 2, Figure 3), including the KOOS, IKDC, Tegner, Lysholm scores and pain VAS. Both the F‐MMA and TSS significantly improved all clinical scores (Lysholm, Tegner, pain VAS, IKDC and KOOS) at 5 years postoperatively in patients with MMPRT (Table 2), whereas the F‐MMA and TSS groups showed no significant differences in pre‐ and postoperative clinical scores.

**Table 1:** Preoperative patient demographics.

Number of patients	43
Gender, men/women	10/33
Age (years)	63.1 ± 7.4 (46–74)
Height (m)	1.58 ± 0.09 (1.41–1.79)
Weight (kg)	66.5 ± 15.6 (48–119)
Body mass index (kg/m^2^)	26.5 ± 4.1 (19.4–38.1)
Root tear classification, type 1/2/3/4/5	4/34/1/4/0
Duration from injury to surgery (days, *n* = 33)	92.1 ± 74.4 (19–276)
Duration from surgery to final follow‐up (years)	5.9 ± 0.8 (5–8)
Femorotibial angle (°)	177.5 ± 1.5 (175–180)
Kellgren–Lawrence, grade 1/2	15/28
Pulllout technique, F‐MMA/two simple stitches	27/16

*Note*: Data are presented as number or mean ± standard deviation (range).

Abbreviation: F‐MMA, FasT‐fix‐dependent modified Mason‐Allen suture.

**Table 2:** Comparison of preoperative and postoperative clinical scores following medial meniscus posterior root repair.

		Preoperative	Postoperative	*p* value
KOOS	Pain	55.6 ± 22.0	86.9 ± 14.6	<0.001*
	Symptoms	66.8 ± 17.0	87.2 ± 10.1	<0.001*
	ADL	68.0 ± 18.3	89.9 ± 10.7	<0.001*
	Sport/Rec	30.0 ± 23.9	65.7 ± 29.0	<0.001*
	QOL	32.3 ± 16.8	72.0 ± 21.9	<0.001*
IKDC score		39.4 ± 14.9	71.2 ± 17.0	<0.001*
Lysholm score		61.7 ± 10.2	90.3 ± 7.2	<0.001*
Tegner activity score		1.7 ± 1.0	3.3 ± 0.8	<0.001*
Pain visual analogue scale		41.9 ± 25.9	8.7 ± 15.4	<0.001*

*Note*: Data are expressed as mean ± standard deviation. Statistical differences between the preoperative and postoperative data were analysed using the Wilcoxon signed‐rank test.

Abbreviations: ADL, activities of daily living; IKDC, International Knee Documentation Committee; KOOS, Knee Injury and Osteoarthritis Outcome Score; QOL, knee‐related quality of life; Sport/Rec, sports and recreational function.

* *p* < 0.001.

However, the progression of OA cannot be completely suppressed. The KL grades at the final follow‐up, with a mean duration of 5.9 years, were as follows: KL grade 1, 2 cases; KL grade 2, 19 cases; and KL grade 3, 22 cases. No knees showed complete disappearance of the joint space; in other words, there was no KL grade of 4. During the follow‐up period, the rate of subsequent knee‐related surgical treatment was 11.6% in pullout‐repaired knees and 7.0% in contralateral knees. One patient sustained an anterior cruciate ligament tear in the ipsilateral knee. This case was not included in the above percentage, as conservative treatment was selected because of the patient's age (63 years) and low activity levels. Postoperative surgeries other than second‐look arthroscopy and plate/screw removal included arthroscopic debridement for arthrofibrosis with a limited range of motion (one case), additional all‐inside suture repair during second‐look arthroscopy for poor healing (two cases), and partial meniscectomy during second‐look arthroscopy for flap tears (without symptoms, two cases; Table 3). None of the 43 patients required ipsilateral knee arthroplasty during the follow‐up, and the survival rate after pullout repair was 100%. Three patients required knee‐related surgery on the contralateral knee: one underwent pullout repair for MMPRT, whereas the other two required unicompartmental knee arthroplasty and total knee arthroplasty because of severe osteoarthritis.

**Table 3:** Postoperative knee surgeries other than second‐look arthroscopy and screw/plate removal.

	Ipsilateral knee	Contralateral knee
Arthroscopic debridement (arthrofibrosis)	1	0
MM partial meniscectomy (flap tear)	2	0
MM repair (poor healing)	2	0
Transtibial pullout repair (MMPRT)	0	1
Unicompartmental knee arthroplasty	0	1
Total knee arthroplasty	0	1
Total	5 (11.6%)	3 (7.0%)

Abbreviations: MM, medial meniscus; MMPRT, medial meniscus posterior root tear.

## DISCUSSION

This study demonstrated that all parameters of the 5‐year postoperative clinical outcomes in both the F‐MMA and TSS cohorts significantly improved compared with those in the preoperative cohorts. Furthermore, there was no progression to KL grade 4 or conversion to arthroplasty in the knees treated with pullout repair. Therefore, our hypothesis is confirmed.

The results of our study are consistent with those of previous studies with mid‐ to long‐term follow‐up after MMPR pullout repair, demonstrating comparable or even superior outcomes. Krych et al. followed 47 patients (21 men and 26 women) with degenerative MMPRT nonoperatively for a mean of 14 years (range, 11–18 years) [22]. At the final follow‐up, 25 patients (53%) had progressed to total knee arthroplasty, 8 patients (17%) had died, and 14 patients (30%) had not progressed to total knee arthroplasty. At a minimum 10‐year follow‐up, 37 of 39 living patients (95%) had failed nonoperative treatment. A short‐term study also demonstrated good results, showing that the F‐MMA pullout repair improved all clinical evaluation scores in 32 patients who underwent MMPR repair, with a mean follow‐up of 36.1 months. The F‐MMA pullout repair yields satisfactory clinical outcomes [10]. The demographic data of patients in this study were slightly different from those in other studies, including differences in age (63.1 years) and BMI (26.5 kg/m^2^). However, the clinical outcomes of this study were comparable to those of previous studies [10, 22].

Several studies have reported survival rates using arthroplasty as an endpoint. Bernard et al. reported a survival rate of 100% in a cohort of 15 patients (10 women and 5 men) with an average age of 46.1 years and BMI 32.0, followed for 5 years [4]. Chung et al. reported a survival rate of 97.3% in a cohort of 37 patients (32 women and 5 men) with an average age of 56.8 years and BMI of 26.2, followed for 5 years, and 79.6% at 10 years [6]. In the latter study, the survival rate decreased over time, underscoring the need for long‐term follow‐up of the patients included in this study. Chung et al. included patients from 2005 to 2009 [6], Krych et al. included those from 2005 to 2013 [22] and Bernard et al. from 2005 to 2016 [4]. Patients treated between 2016 and 2018 were included in this study. Between 2012 and 2015, risk factors leading to poor clinical outcomes were reported, with findings indicating that chondral lesions, such as Outerbridge grades 3 or 4 and varus alignment >5°, were independent predictors of inferior clinical outcomes [1, 28]. Therefore, by excluding patients with these risk factors and implementing more stringent patient selection criteria, we believe that excellent clinical outcomes and 100% joint preservation rates were achieved in the present study.

The minimal clinically important difference (MCID) threshold values for each subjective score at 2 years after arthroscopic meniscal repair were reported to be 10.9, 12.3, 11.8, 11.4, 16.7 and 16.9 for the IKDC score, KOOS symptoms, pain, ADL, sport and QOL subscales, respectively [27]. In this study, at 5 years postoperatively, all clinical scores showed improvements that exceeded the MCID thresholds reported in a previous study (Table 2). The patient‐acceptable symptomatic state (PASS) values for each subjective score at 2 years postoperatively in the same study were 69.0, 75.0, 80.6, 92.6, 80.0 and 56.3, for the IKDC score and KOOS symptoms, pain, ADL, sports and QOL subscales, respectively [27]. Thus, the 5‐year postoperative KOOS ADL and Sport/Rec subscale scores in the present study were lower than the PASS values determined in a previous study (Table 2). It should be noted that the previous study [27] involved a much younger cohort than that in this study, with an average age of 34.0 (range, 19.5–49.5) years. Moreover, the KOOS Sport/Rec subscale includes questions on sports activities such as jumping and running. Regarding ADL, due to cultural practices and daily activities in our region (Asia), deep knee flexion (such as in *seiza* sitting) is often required at home, which may contribute to relatively lower KOOS ADL scores.

This study had several limitations. First, this was a retrospective study conducted without blinding for the surgical techniques. Second, the sample size was relatively small and may only be applicable to Asian populations. Third, given the good clinical outcomes and high levels of satisfaction among all patients 5 years postoperatively, radiologic evaluations such as MRI examinations performed to assess joint space narrowing, progression of knee OA, and the status of the MMPR in every case would have been impractical from a healthcare economics perspective. Therefore, MRI evaluations were not performed, and MM extrusion was not assessed at the final follow‐up. This omission represents a major limitation of the present study, especially given that factors such as age, BMI, healing status and alignment have been reported to correlate with MM extrusion [16, 31]. Additionally, the tibial tunnel position was not evaluated. Accurate anatomic placement of the tibial tunnel has been shown to delay the progression of medial joint space narrowing [17], highlighting another limitation of the study. Finally, the minimum 5‐year postoperative follow‐up period may be too short to evaluate clinical outcomes and subsequent knee‐related surgical treatments following pullout repair in patients with MMPRT.

## CONCLUSION

This study demonstrated that both F‐MMA and TSS pullout repairs yielded satisfactory clinical outcomes in patients with MMPRT with a mean follow‐up of 5.9 years, and that no conversion to knee arthroplasty was required. However, the progression of OA could not be completely suppressed, although there were no KL grade 4 cases, and subsequent knee‐related surgical treatments were necessary in 11.6% of repaired knees during the follow‐up period. Further follow‐up is warranted to assess long‐term survival rates.

## AUTHOR CONTRIBUTIONS

Takayuki Furumatsu designed this study. Material preparation, data collection and analysis were performed by Kazuhisa Sugiu, Yuki Okazaki and Yusuke Kamatsuki. The first draft of the manuscript was written by Yuki Okazaki, and all authors commented on previous versions of the manuscript. All authors read and approved the final manuscript.

## CONFLICT OF INTEREST STATEMENT

The authors declare no conflicts of interest.

## ETHICS STATEMENT

This study was conducted in accordance with the principles of the Declaration of Helsinki. The study was approved by the Ethics Committee of Okayama University (No. 1857). Written informed consent was obtained from all patients.

## Data Availability

The data that support the findings of this study are available from the corresponding author, upon reasonable request.

## References

[1] Ahn JH , Jeong HJ , Lee YS , Park JH , Lee JW , Park JH , et al. Comparison between conservative treatment and arthroscopic pull‐out repair of the medial meniscus root tear and analysis of prognostic factors for the determination of repair indication. Arch Orthop Trauma Surg. 2015;135:1265–1276. 26142540 10.1007/s00402-015-2269-8

[2] Allaire R , Muriuki M , Gilbertson L , Harner CD . Biomechanical consequences of a tear of the posterior root of the medial meniscus. Similar to total meniscectomy. J Bone Joint Surg Am Vol. 2008;90:1922–1931. 10.2106/JBJS.G.00748 18762653

[3] Bae JH , Paik NH , Park GW , Yoon JR , Chae DJ , Kwon JH , et al. Predictive value of painful popping for a posterior root tear of the medial meniscus in middle‐aged to older Asian patients. Arthrosc J Arthrosc Rel Surg. 2013;29:545–549. 10.1016/j.arthro.2012.10.026 23375180

[4] Bernard CD , Kennedy NI , Tagliero AJ , Camp CL , Saris DBF , Levy BA , et al. Medial meniscus posterior root tear treatment: a matched cohort comparison of nonoperative management, partial meniscectomy, and repair. Am J Sports Med. 2020;48:128–132. 31765234 10.1177/0363546519888212

[5] Chung KS , Ha JK , Ra HJ , Kim JG . Preoperative varus alignment and postoperative meniscus extrusion are the main long‐term predictive factors of clinical failure of meniscal root repair. Knee Surg Sports Traumatol Arthrosc. 2021;29:4122–4130. 33730189 10.1007/s00167-020-06405-7

[6] Chung KS , Ha JK , Ra HJ , Yu WJ , Kim JG . Root repair versus partial meniscectomy for medial meniscus posterior root tears: comparison of long‐term survivorship and clinical outcomes at minimum 10‐year follow‐up. Am J Sports Med. 2020;48:1937–1944. 32437216 10.1177/0363546520920561

[7] Fujii M , Furumatsu T , Xue H , Miyazawa S , Kodama Y , Hino T , et al. Tensile strength of the pullout repair technique for the medial meniscus posterior root tear: a porcine study. Int Orthop. 2017;41:2113–2118. 28707050 10.1007/s00264-017-3561-8

[8] Furumatsu T , Hiranaka T , Okazaki Y , Kintaka K , Kodama Y , Kamatsuki Y , et al. Medial meniscus posterior root repairs: a comparison among three surgical techniques in short‐term clinical outcomes and arthroscopic meniscal healing scores. J Orthop Sci. 2022;27:181–189. 33581924 10.1016/j.jos.2020.11.013

[9] Furumatsu T , Miyazawa S , Fujii M , Tanaka T , Kodama Y , Ozaki T . Arthroscopic scoring system of meniscal healing following medial meniscus posterior root repair. Int Orthop. 2019;43:1239–1245. 30069591 10.1007/s00264-018-4071-z

[10] Furumatsu T , Miyazawa S , Kodama Y , Kamatsuki Y , Okazaki Y , Hiranaka T , et al. Clinical outcomes of medial meniscus posterior root repair: a midterm follow‐up study. Knee. 2022;38:141–147. 36058121 10.1016/j.knee.2022.08.010

[11] Furumatsu T , Okazaki Y , Okazaki Y , Hino T , Kamatsuki Y , Masuda S , et al. Injury patterns of medial meniscus posterior root tears. Orthop Traumatol Surg Res. 2019;105:107–111. 30442555 10.1016/j.otsr.2018.10.001

[12] Itou J , Kuwashima U , Itoh M , Okazaki K . High tibial osteotomy for medial meniscus posterior root tears in knees with moderate varus alignment can achieve favorable clinical outcomes. J Exp Orthop. 2022;9:65. 35796797 10.1186/s40634-022-00504-9 PMC9263016

[13] Jiang EX , Abouljoud MM , Everhart JS , DiBartola AC , Kaeding CC , Magnussen RA , et al. Clinical factors associated with successful meniscal root repairs: a systematic review. Knee. 2019;26:285–291. 30772183 10.1016/j.knee.2019.01.005

[14] Kamatsuki Y , Furumatsu T , Hiranaka T , Okazaki Y , Kintaka K , Kodama Y , et al. Epidemiological features of acute medial meniscus posterior root tears. Int Orthop. 2023;47:2537–2545. 37329453 10.1007/s00264-023-05848-0 PMC10522759

[15] Kamatsuki Y , Furumatsu T , Hiranaka T , Okazaki Y , Kodama Y , Kintaka K , et al. Accurate placement of a tibial tunnel significantly improves meniscal healing and clinical outcomes at 1 year after medial meniscus posterior root repair. Knee Surg Sports Traumatol Arthrosc. 2021;29:3715–3723. 33388829 10.1007/s00167-020-06376-9

[16] Kawada K , Furumatsu T , Yokoyama Y , Higashihara N , Tamura M , Ozaki T . Meniscal healing status after medial meniscus posterior root repair negatively correlates with a midterm increase in medial meniscus extrusion. Knee Surg Sports Traumatol Arthrosc. 2024;32:2219–2227. 38741370 10.1002/ksa.12245

[17] Kawada K , Okazaki Y , Tamura M , Yokoyama Y , Ozaki T , Furumatsu T . Accurate tibial tunnel position in transtibial pullout repair for medial meniscus posterior root tears delays the progression of medial joint space narrowing. Knee Surg Sports Traumatol Arthrosc. 2024;32:2023–2031. 38747021 10.1002/ksa.12229

[18] Kellgren JH , Lawrence JS . Radiological assessment of osteo‐arthrosis. Ann Rheum Dis. 1957;16:494–502. 13498604 10.1136/ard.16.4.494 PMC1006995

[19] Kim C , Bin SI , Kim JM , Lee BS , Kim TH . Progression of radiographic osteoarthritis after partial meniscectomy in degenerative medial meniscal posterior root tears was greater in varus‐ than in neutral‐aligned knees: a minimum 5‐year follow‐up. Knee Surg Sports Traumatol Arthrosc. 2020;28:3443–3449. 32067077 10.1007/s00167-020-05905-w

[20] Kim YM , Joo YB , Lee WY , Kim YK . Remodified Mason‐Allen suture technique concomitant with high tibial osteotomy for medial meniscus posterior root tears improved the healing of the repaired root and suppressed osteoarthritis progression. Knee Surg Sports Traumatol Arthrosc. 2021;29:1258–1268. 32712682 10.1007/s00167-020-06151-w

[21] Kodama Y , Furumatsu T , Fujii M , Tanaka T , Miyazawa S , Ozaki T . Pullout repair of a medial meniscus posterior root tear using a FasT‐Fix ® all‐inside suture technique. Orthop Traumatol Surg Res. 2016;102:951–954. 27567426 10.1016/j.otsr.2016.06.013

[22] Krych AJ , Lamba A , Wang AS , Boos AM , Camp CL , Levy BA , et al. Nonoperative management of degenerative medial meniscus posterior root tears: poor outcomes at a minimum 10‐year follow‐up. Am J Sports Med. 2023;51:2603–2607. 37434486 10.1177/03635465231185132

[23] LaPrade CM , James EW , Cram TR , Feagin JA , Engebretsen L , LaPrade RF . Meniscal root tears: a classification system based on tear morphology. Am J Sports Med. 2015;43:363–369. 25451789 10.1177/0363546514559684

[24] Lee DW , Lee SH , Kim JG . Outcomes of medial meniscal posterior root repair during proximal tibial osteotomy. Is root repair beneficial? Arthrosc J Arthrosc Rel Surg. 2020;36:2466–2475. 10.1016/j.arthro.2020.04.038 32389775

[25] Lee HI , Park D , Cho J . Clinical and radiological results with second‐look arthroscopic findings after open wedge high tibial osteotomy without arthroscopic procedures for medial meniscal root tears. Knee Surg Rel Res. 2018;30:34–41. 10.5792/ksrr.17.035 PMC5853171 29482302

[26] Lee OS , Lee SH , Lee YS . Comparison of the radiologic, arthroscopic, and clinical outcomes between repaired versus unrepaired medial meniscus posterior horn root tear during open wedge high tibial osteotomy. J Knee Surg. 2021;34:057–066. 10.1055/s-0039-1692992 31288272

[27] Maheshwer B , Wong SE , Polce EM , Paul K , Forsythe B , Bush‐Joseph C , et al. Establishing the minimal clinically important difference and patient‐acceptable symptomatic state after arthroscopic meniscal repair and associated variables for achievement. Arthrosc J Arthrosc Rel Surg. 2021;37:3479–3486. 10.1016/j.arthro.2021.04.058 33964390

[28] Moon HK , Koh YG , Kim YC , Park YS , Jo SB , Kwon SK . Prognostic factors of arthroscopic pull‐out repair for a posterior root tear of the medial meniscus. Am J Sports Med. 2012;40:1138–1143. 22316547 10.1177/0363546511435622

[29] Moon HS , Choi CH , Jung M , Lee DY , Hong SP , Kim SH . Early surgical repair of medial meniscus posterior root tear minimizes the progression of meniscal extrusion: 2‐year follow‐up of clinical and radiographic parameters after arthroscopic transtibial pull‐out repair. Am J Sports Med. 2020;48:2692–2702. 32730732 10.1177/0363546520940715

[30] Nha KW , Lee YS , Hwang DH , Kwon JH , Chae DJ , Park YJ , et al. Second‐look arthroscopic findings after open‐wedge high tibia osteotomy focusing on the posterior root tears of the medial meniscus. Arthrosc J Arthrosc Rel Surg. 2013;29:226–231. 10.1016/j.arthro.2012.08.027 23369476

[31] Nie S , Li H , Liao X , Liu Q , Lan M . Younger patients, lower BMI, complete meniscus root healing, lower HKA degree and shorter preoperative symptom duration were the independent risk factors correlated with the good correction of MME in patients with repaired MMPRTs. Knee Surg Sports Traumatol Arthrosc. 2023;31:3775–3783. 36790456 10.1007/s00167-023-07330-1

[32] Okazaki Y , Furumatsu T , Hiranaka T , Kodama Y , Kamatsuki Y , Kintaka K , et al. Two simple stitches for medial meniscus posterior root repair prevents the progression of meniscal extrusion and reduces intrameniscal signal intensity better than modified Mason‐Allen sutures. Eur J Orthop Surg Traumatol. 2021;31:1005–1013. 33219860 10.1007/s00590-020-02830-z

[33] Okazaki Y , Furumatsu T , Okazaki Y , Masuda S , Hiranaka T , Kodama Y , et al. Medial meniscus posterior root repair decreases posteromedial extrusion of the medial meniscus during knee flexion. Knee. 2020;27:132–139. 31882388 10.1016/j.knee.2019.09.005

[34] Okazaki Y , Furumatsu T , Yamaguchi T , Kodama Y , Kamatsuki Y , Masuda S , et al. Medial meniscus posterior root tear causes swelling of the medial meniscus and expansion of the extruded meniscus: a comparative analysis between 2D and 3D MRI. Knee Surg Sports Traumatol Arthrosc. 2020;28:3405–3415. 31243505 10.1007/s00167-019-05580-6

[35] Okazaki Y , Furumatsu T , Yamauchi T , Okazaki Y , Kamatsuki Y , Hiranaka T , et al. Medial meniscus posterior root repair restores the intra‐articular volume of the medial meniscus by decreasing posteromedial extrusion at knee flexion. Knee Surg Sports Traumatol Arthrosc. 2020;28:3435–3442. 32253480 10.1007/s00167-020-05953-2

[36] Padalecki JR , Jansson KS , Smith SD , Dornan GJ , Pierce CM , Wijdicks CA , et al. Biomechanical consequences of a complete radial tear adjacent to the medial meniscus posterior root attachment site: in situ pull‐out repair restores derangement of joint mechanics. Am J Sports Med. 2014;42:699–707. 24585675 10.1177/0363546513499314

[37] Potter HG , Linklater JM , Allen AA , Hannafin JA , Haas SB . Magnetic resonance imaging of articular cartilage in the knee. An evaluation with use of fast‐spin‐echo imaging. J Bone Joint Surg. 1998;80:1276–1284. 9759811 10.2106/00004623-199809000-00005

[38] Ridley TJ , Ruzbarsky JJ , Dornan GJ , Woolson TE , Poulton RT , LaPrade RF , et al. Minimum 2‐year clinical outcomes of medial meniscus root tears in relation to coronal alignment. Am J Sports Med. 2022;50:1254–1260. 35420502 10.1177/03635465221080167

